# Development and evaluation of an immuno-MALDI (iMALDI) assay for angiotensin I and the diagnosis of secondary hypertension

**DOI:** 10.1186/1559-0275-10-20

**Published:** 2013-12-20

**Authors:** Alexander G Camenzind, Jessica Grace van der Gugten, Robert Popp, Daniel T Holmes, Christoph H Borchers

**Affiliations:** 1 Genome British Columbia Proteomics Centre, University of Victoria, 3101-4464 Markham St, Victoria, BC V8Z 7X8, Canada; 2 Department of Pathology and Laboratory Medicine, St. Paul’s Hospital, University of British Columbia, 1081 Burrard Street, Vancouver, BC V6Z 1Y6, Canada; 3 Department of Biochemistry and Microbiology, University of Victoria, Victoria, BC V8P 5C2, Canada

**Keywords:** Hypertension, Angiotensin-I, Plasma renin activity, PRA, iMALDI, Immuno-MALDI

## Abstract

Plasma renin activity (PRA) is an essential analytical tool for screening and diagnosis of secondary forms of hypertension. Typically, PRA is measured by competitive radioimmunoassay, but there are significant drawbacks to this technique including non-specificity, long analysis times, narrow calibration range, and the requirement for radionucleotides. In this paper, we report a method for plasma renin activity determination by immuno-MALDI mass spectrometry detection. This method overcomes the issues of non-specificity and long analytical times present with RIA, and does not require the use of radionucleotides. As an initial methodological evaluation, plasma renin activity results obtained by radioimmunoassay, LC/ESI-MS/MS, and immuno-MALDI on 64 samples from an outpatient primary aldosteronism screening program have been compared. A strong correlation was found between immuno-MALDI and radioimmunoassay (R^2^ = 0.9412, 62/64 within the 95% CI of the Bland-Altman plot), and iMALDI and LC/ESI-MS/MS (R^2^ = 0.9471, 62/64 within the 95% CI of the Bland-Altman plot). Technical replicates showed a 4.8% CV, while inter- and intra-day replicates showed CVs of 17.3% and 17.2% respectively. We have developed an assay capable of measuring PRA without the use of radionucleotides. This immuno-MALDI approach affords the specificity of MS while avoiding the long analytical run times and technical problems associated with HPLC. With the use of robotic sample preparation to optimize precision, this assay should be adaptable to clinical environments.

## Background

Hypertension is a world-wide epidemic affecting more than 1 billion people and causing 7.1 million deaths per year [1]. The consequences of untreated hypertension include increased risk of: coronary and peripheral vascular disease, stroke, congestive heart failure, and chronic renal impairment. Early identification of treatable and/or curable forms of hypertension is therefore critical to the prevention of the associated chronic diseases.

Arterial blood pressure is regulated by the renin-angiotensin-aldosterone system (RAAS). Dysregulation of this system leads to the development of certain hypertensive states that are responsive to surgical, vascular, or medical intervention–most notably primary aldosteronism (PA) and renal arterial stenosis. The RAAS is regulated by renin, a proteolytic enzyme secreted by the juxtaglomerular cells of the renal glomerulus in response to β-1 adrenergic stimulation, decreased arterial blood pressure, and decreased delivery of NaCl to the macula densa. Renin cleaves the decapeptide angiotensin-I (Ang-I) from angiotensinogen, an α-2 globulin constitutively manufactured by the liver. Through the action of the angiotensin-converting-enzyme (ACE), Ang-I is converted to angiotensin-II (Ang-II), a potent vasoconstrictor. This leads to a rapid rise in blood pressure and subsequent upregulation of aldosterone production by the adrenal glands. Both Ang-II and aldosterone then feedback to decrease plasma renin activity (PRA).

The determination of PRA is a critical tool for the screening and diagnostic process of PA and has a long history of diagnostic utility [2,3]. Traditionally, PRA assays have been performed using a 1-3 h generation of Ang-I, which is sometimes extended to 18 h for samples demonstrating very low activity [3]. Early assays used a pH of 7.4 [2] which was later decreased to 5.5 - 6.0 to improve sensitivity [3,4] and angiotensinase inhibition [5]. The Ang-I generated was protected from proteolytic degradation either by adding an ACE/angiotensinase inhibitor [3] or by antibody capture [6] and the generation was halted either chemically (11) or by using an ice-bath [3]. Although various immunoassay techniques for PRA have been used [7,8], radioimmunoassay (RIA) has been the mainstay [2].

Recently, screening efforts for PA have increased as physicians became aware of its high prevalence [9,10]. This has increased the need for high-throughput methods for measuring RAAS components. Accordingly, there has been movement away from traditional PRA assays because they are not readily amenable to automation. In their place, sandwich assays measuring plasma renin concentration (PRC) have become commonplace, particularly since the development of automated chemiluminescent approaches [11-13].

Although some studies have found PRC to be an adequate substitute for PRA when calculating the aldosterone to renin ratio [12,14-17], there are a number of arguments for retaining PRA as the method of choice. First, there is a larger body of evidence for PA screening using PRA, and PRA levels correlate better with plasma Ang-I and Ang-II. Direct comparisons of the aldosterone:PRA *versus* aldosterone:PRC ratios generally show that the former has higher area-under-the-ROC-curve [16,18]. Moreover, aldosterone:PRC may suffer from a higher false-positive rate in women [19].

**Figure 1:**
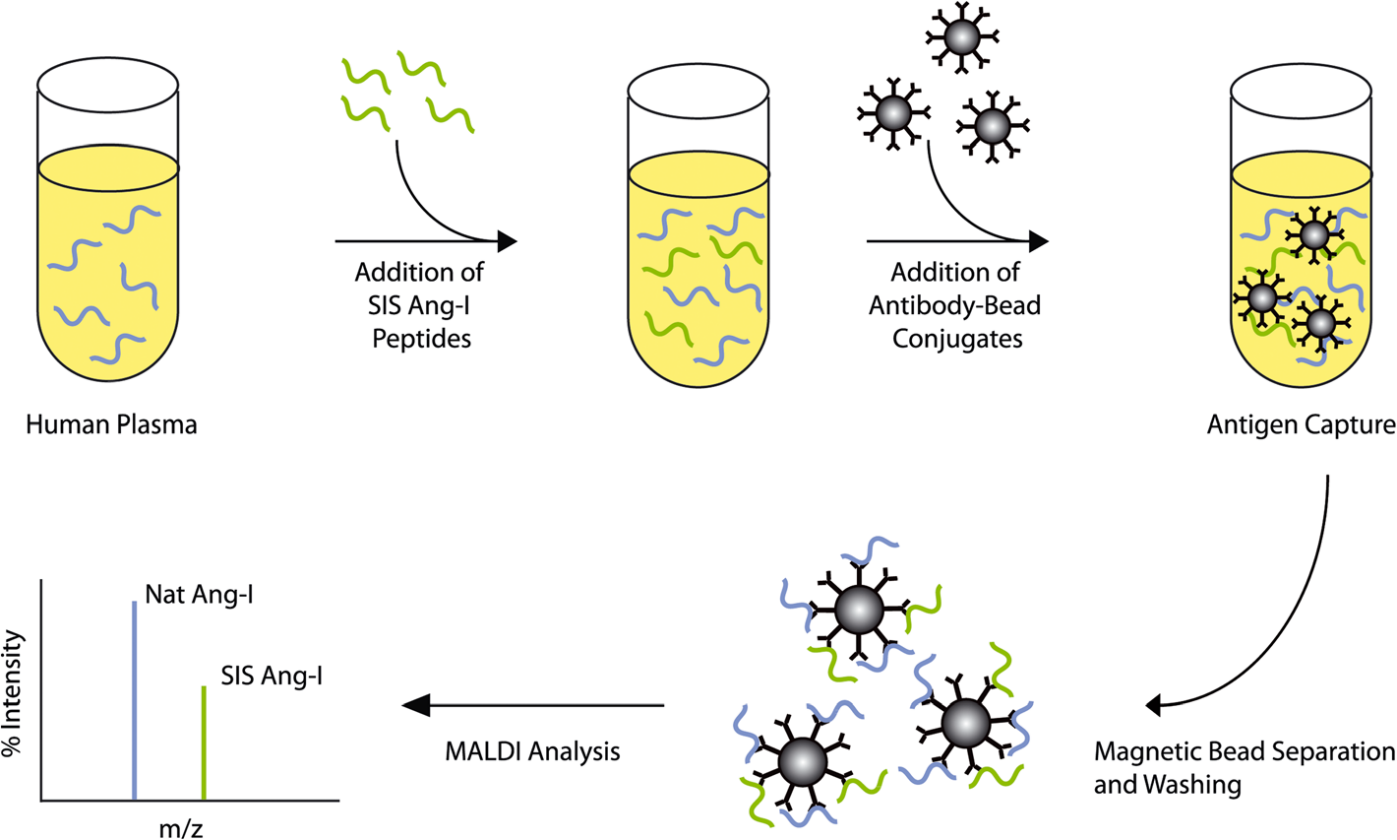
**iMALDI workflow.** Stable-isotope labelled internal standard (SIS)-Ang-I (green) is spiked into plasma and the sample is incubated with anti-Ang-I-antibody conjugated beads. After immunoprecipitation of endogenous (blue) and SIS-Ang-I the beads can be placed directly on a MALDI target. To elute the analytes and permit MALDI-MS analysis, CHCA matrix is applied. The relative abundances of endogenous to SIS-Ang-I are used for quantitation.

Enthusiasm for the PRA assay has therefore continued [20], and MS-based approaches for Ang-I determination have been of great interest because of their specificity. Published MS-based methods have used solid-phase extraction (SPE) and positive ion LC/ESI-MS/MS [21,22]. Although this approach is radionucleotide-free, LC/ESI-MS/MS requires considerable expertise and many clinical laboratories have shied away from this technique because of its technical demands [23]. In contrast, MALDI-TOF is widely used in clinical laboratories for the rapid speciation of bacteria and yeast, because of its speed, ease, robustness, and low cost [24-26]. Because of this, we have developed a PRA assay using a MALDI platform (Figure 1). This assay uses immunocapture coupled to MALDI analysis (iMALDI) [27-30], is free of radionucleotides, does not require HPLC, and shows good correlation with existing clinical RIA and LC/ESI-MS/MS methods.

## Results and discussion

This paper describes a 3-way comparison of methods for determining PRA: the traditional method, using RIA, which involves radionucleotides; an LC-MS/MS method involving on-line HPLC separation and electrospray ionization [31] for quantitation of angiotensin, and an iMALDI method which does not involve either HPLC separation or radioactivity, but instead utilizes antibody capture of angiotensin. In the iMALDI method, the amount of angiotensin present is determined by direct MALDI analysis of the affinity beads which are placed on the MALDI target without prior elution of the captured analyte. The same patient samples were analyzed by all three methods.

### PRA determination by the LC/ESI-MS/MS method

**Figure 2:**
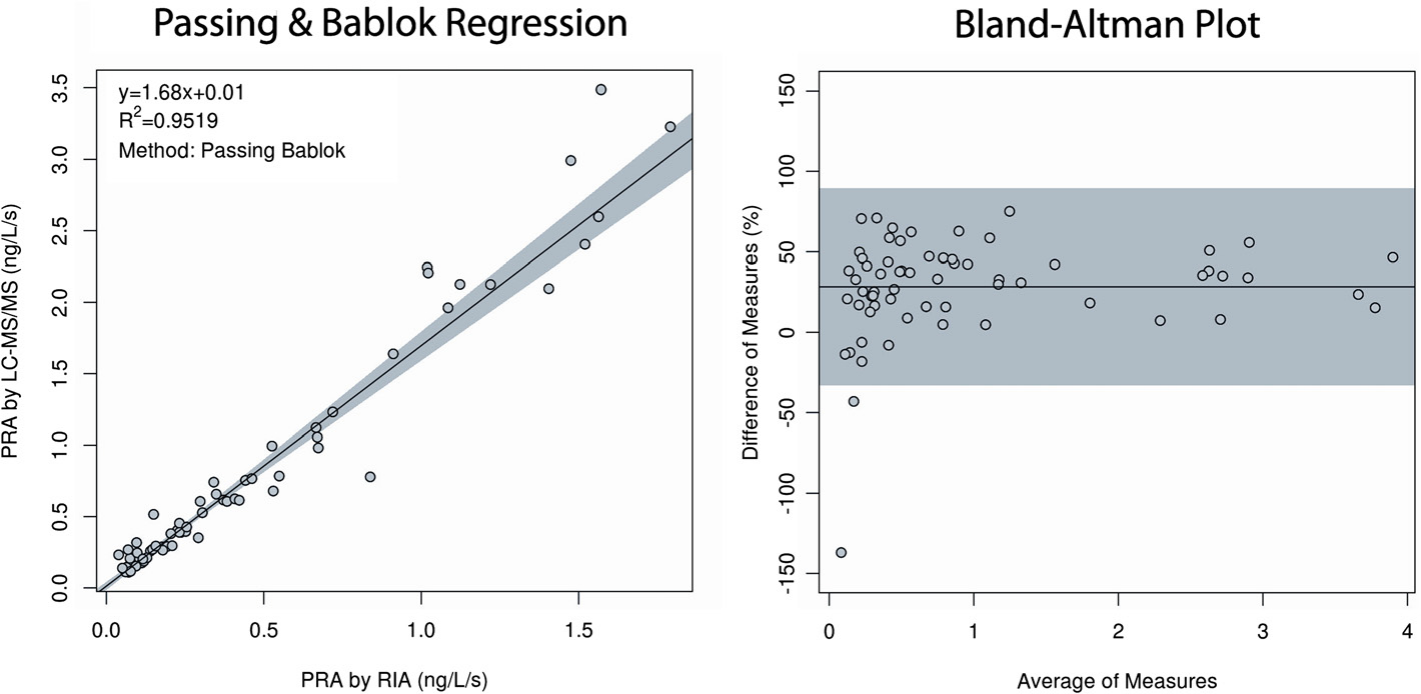
**Correlation of RIA with LC/ESI-MS/MS.** PRA was determined by RIA and LC/ESI-MS/MS for 64 patients. The correlation is shown by Passing and Bablok regression as well as a Bland Altman difference plot. Shaded regions represent a 95% confidence interval.

LC-MS/MS analysis showed a coefficient of determination (R^2^) of 0.9296 for 64 clinical RIA values, with a slope of 1.68. Sixty-two of the 64 samples were within the 95% CI of the Bland-Altman plot (Figure 2). These two methods were used as comparison methods to judge the correlation of the iMALDI results with those from a PRA assay at pH 7.4 (for the RIA analysis) and pH 6.0 (for the LC/ESI-MS/MS analysis). The inter-day reproducibility of biological replicates of the LC/ESI-MS/MS assay was found to be 8.3%, 7.7%, and 9.0% CV at PRA values of 0.26, 1.34, and 5.63 ng/L/s respectively, as determined by a CLSI EP5-A2-compliant protocol [32].

### Comparison of PRA determination by iMALDI, RIA, and LC/ESI-MS/MS

**Figure 3:**
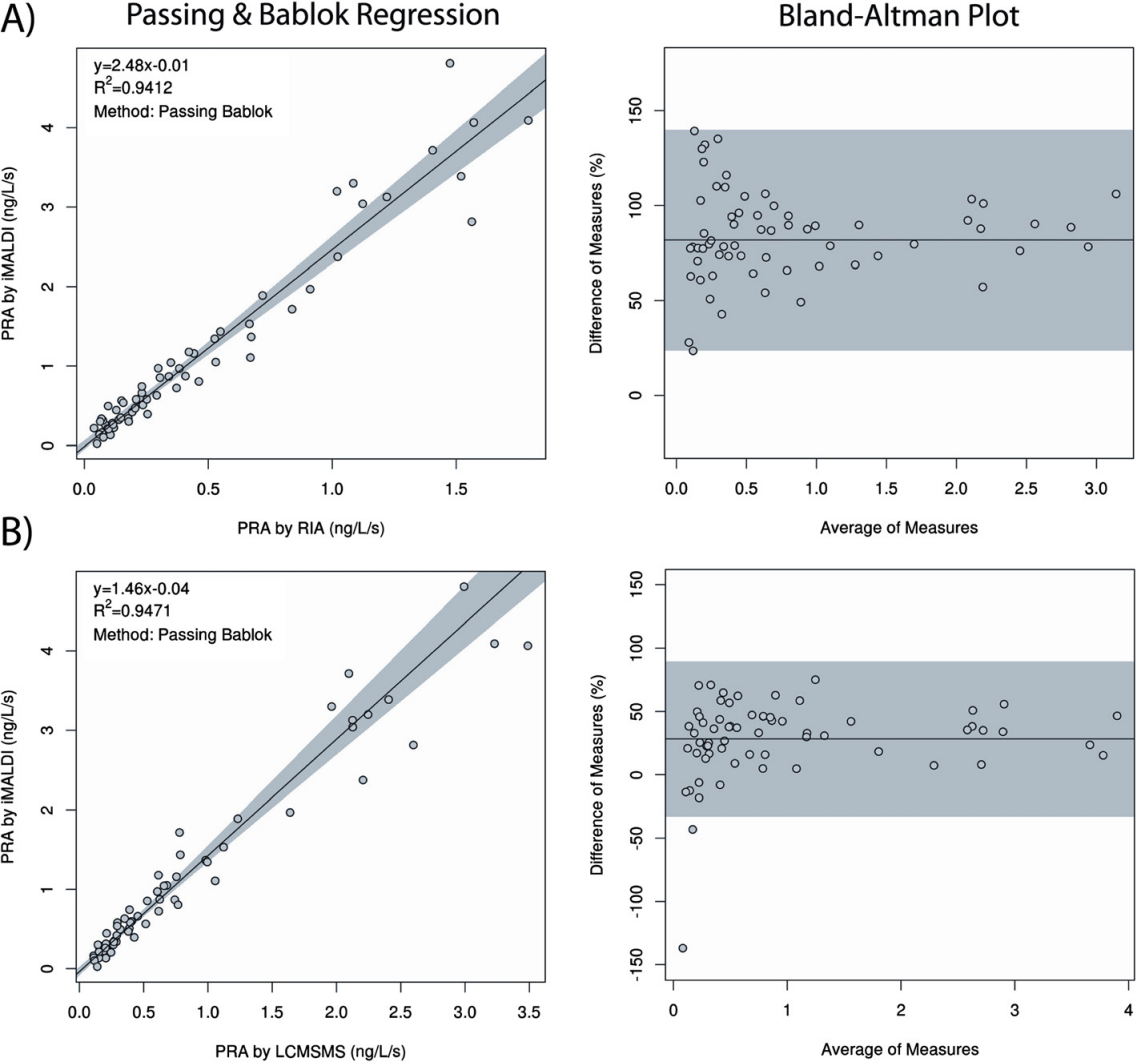
**Correlation of iMALDI with RIA and LC/MS/MS. A)** Difference plot comparisons for RIA and iMALDI. **B)** Difference plot comparison for LC/ESI-MS/MS and iMALDI.

The iMALDI versus RIA values showed an R^2^ of 0.9412 across 64 clinical samples, with a slope of 2.48. Sixty-two of the 64 samples were within the 95% CI of the Bland-Altman plot (Figure 3A). The iMALDI results were also compared to PRA values determined by LC-MS/MS, and showed an R^2^ of 0.9471 with a slope of 1.46. Sixty two of the 64 samples were within the 95% CI of the Bland-Altman plot (Figure 3B). Inter-day (1 replicate per day for 5 consecutive days)- and intra-day (5 replicates in 1 day) reproducibility of biological replicates measured by iMALDI were 17.3% CV and 17.2% CV, respectively, at a PRA value of 0.38 ng/L/s, with technical replicates averaging 4.8% CV.

**Figure 4:**
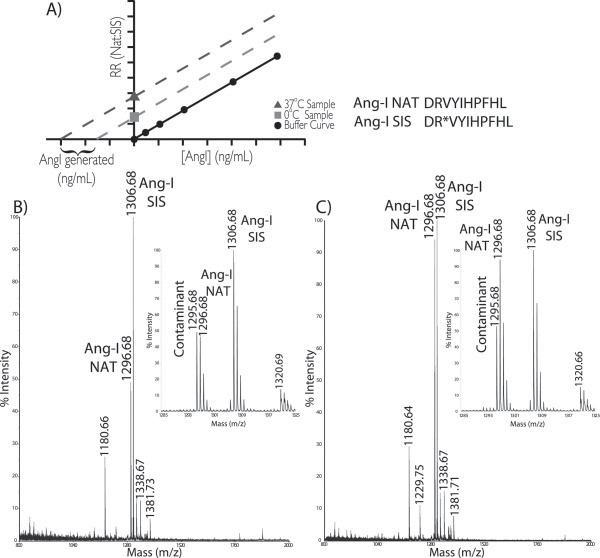
**The determination of PRA by iMALDI. A)** A standard curve is created from antibody captures of natural and SIS peptides. **B)** MALDI spectra showing capture of natural and SIS Ang-I in a 4°C sample. **C)** MALDI spectra showing capture of natural and SIS Ang-I in a 37°C sample.

The iMALDI assay for PRA using a 1-h incubation and a 1-h antibody capture which shows a strong correlation with RIA and LC-MS/MS methods (n = 64, R^2^ = 0.9412 and R^2^ = 0.9471 with RIA and LC-MS/MS, respectively). This iMALDI method improves on many aspects of the RIA procedure -- mainly the exclusion of radionucleotides and inaccuracies associated with antibody cross-reactivity. This method employs antigen-concentration by immunocapture, and direct elution on the MALDI target (Figure 1), which reduces the risk of non-specific binding to plastics [33]. The specificity provided by the mass spectrometer allows the accurate determination of MWs of all antibody-captured analytes, ensuring that antibody cross-reactivity does not result in analytical interferences. This specificity is important, because some between-method variability is believed to result from biologically-inactive, yet immunologically cross-reactive components present in patient plasma [34]. In iMALDI, if additional components are captured by the antibody, they are detected as different components and can be accounted for (Figure 4).

In MALDI-MS, a standard curve can be created by capture of synthetic natural and SIS Ang-I peptide from 1xPBS/0.03% CHAPS buffer. The same standard curve is used for both the generated (37°C) and blank (4°C) samples to quantitate Ang-I and to calculate PRA. The use of SIS peptides as internal standard allows MALDI, and our iMALDI approach, to be quantitative [35].

PRA values determined by iMALDI were compared to those of RIA and LC/ESI-MS/MS. Figure 3A shows the regression relationship of iMALDI to RIA for PRA, which has a slope of 2.48. The deviation of the slope from unity can be partially explained by the substantial increase in PRA seen for incubations performed at lower pH [36]. Historically, PRA assays were performed at physiological pH = 7.4, but this was later altered to pH = 6.0 to increase analytical sensitivity and improve angiotensinase inhibition [3]. When identical samples are run at pH = 7.4 and pH = 6.0, the PRA for samples run at pH = 6.0 show roughly twice the activity of the samples run at pH = 7.4 [3]. The RIA assay utilized pH = 7.4, while the iMALDI assay used pH = 6.0. Additionally, there are slight differences observed in PRA values when plasma samples are diluted to different extents [36]. Figure 3B shows the comparison of iMALDI with LC/ESI-MS/MS, which has a slope of 1.46. Both methods were run at pH = 6.0, but the LC/ESI-MS/MS method has an Ang-I generation time of 3 h, compared to a 1-h generation for the iMALDI assay. Because Ang-I generation by renin is substrate dependant, different generation times will result in different numerical PRA results with shorter incubations resulting in higher values [37].

## Conclusions

We have developed an iMALDI assay for PRA that eliminates the use of radionucleotides and reduces the deleterious effects of antibody cross-reactivity. The iMALDI results using this assay show a strong correlation with existing PRA methods currently used in routine clinical care, while requiring only a 1-h Ang-I generation period and a 1-h antigen capture step. With automation of sample preparation, we expect to see even further improvements in the throughput and precision of the assay. MALDI instruments are currently being installed in clinical laboratories throughout the world for the identification of bacteria [38]. Because iMALDI can be performed on these same instruments, the assay presented here has potential for translation to the clinic.

## Experimental procedures

### Plasma samples

Plasma samples were obtained from a cohort of 64 subjects from the outpatient PRA-screening program of St. Paul’s Hospital (SPH) Laboratory, selected to span the analytical range of the RIA assay (0.05-2.00 ng/L/s using pH = 7.4 incubation) [6]. Samples were collected into pre-chilled EDTA tubes, centrifuged at 4°C, and frozen at −20°C for at most 10 days. Specimens were thawed for 5 min in a room temperature (RT) water bath and immersed in ice-water prior to RIA [6] and LC/ESI-MS/MS analysis, using a variation of our previous method [31]. Samples were then refrozen and maintained at −80°C until iMALDI analysis at the University of Victoria (UVic) - Genome BC Proteomics Centre. Because freeze-thaw cycles can cause elevation of PRA due to prorenin cryoactivation, samples spent minimal times at refrigeration temperatures where cryoactivation is greatest [3].

This study was approved by the research ethics board of the University of British Columbia and SPH.

### Chemicals and reagents

#### RIA and LC/ESI-MS/MS

RIA analysis used a rabbit polyclonal anti-angiotensin antiserum prepared according to a published protocol [39]. Purified Ang-I was purchased from Proteochem. BSA and glacial acetic acid were purchased from EMD Millipore. Radiolabeled Ang-I (Human, [^125^I]Tyr4-, 10 μCi(370 kBq), ^125^I-Ang-I) tracer was purchased from Perkin Elmer. Tris, EDTA, formic acid (FA) and PMSF were purchased from Sigma Aldrich. Dextran was purchased from MP Biomedical, and activated charcoal and methanol were purchased from Fisher Scientific. Stable-isotope labelled internal standard (SIS) Ang-I (DR*VYIHPFHL, +10 Da) was prepared by the UVic - Genome BC Proteomics Centre. Briefly, C-terminal [^13^C]/[^15^N] labeled tryptic peptides were synthesized using an N-(9-fluorenyl)methoxycarbonyl) procedure on a Prelude peptide synthesizer (Protein Technologies). Peptides were purified by HPLC, purity was confirmed by MALDI-TOF-MS, characterization was done by capillary zone electrophoresis at the University of British Columbia (Vancouver, Canada), and by amino acid analysis at the Hospital for Sick Children (Toronto, Canada) [40].

#### iMALDI reagents

Goat polyclonal anti-angiotensin antibody (pAb) (SC-7419) was purchased from Santa Cruz Biotechnology. Magnetic protein G Dynabeads were purchased from Invitrogen. Synthetic peptides (natural and SIS) were synthesized in house [40]. TFA was purchased from Fisher Scientific. PBS, CHAPS, EDTA, maleic acid, neomycin trisulphate salt hydrate, PMSF, ammonium bicarbonate, α-cyano-4-hydroxycinnamic acid (CHCA), ammonium citrate dibasic, LC/MS-grade H_2_O, and LC/MS-Grade ACN were purchased from Sigma Aldrich.

### RIA procedure

#### Preparation of reference standards

Reference standards were prepared by dissolving purified Ang-I in a buffer containing 1% BSA in 0.1 M Tris, to make a stock concentration of 5000 μg/mL Ang-I. The stock solution was spiked and then serially diluted to make a calibration curve at 10, 5, 2.5, 1.25, 0.625, 0.313, and 0.156 ng/mL in a 1% BSA in 0.1 M Tris buffer.

#### Generation of angiotensin I

Frozen plasma samples were thawed for 5 minutes in a RT water bath followed immediately by immersion in an ice-water bath, where they remained, except for the 37°C generation step. Patient samples, reference standards, and controls were aliquoted in duplicate for the 37°C incubation and the blank (ice-water bath) incubation. Ten μL of rabbit anti-angiotensin in a 1 M Tris/0.2 M EDTA buffer at pH = 7.4 were added to each conical polystyrene tube (Evergreen Scientific), followed by 50 μL of standards and unknowns. The 37°C-incubation tubes were placed in a 37°C water bath for exactly 1 h; the blank tubes were kept in the ice bath. At the end of the incubation period, the 37°C samples were immediately returned to the ice bath.

#### RIA analysis

The ^125^I-Ang-I tracer was diluted in 0.1 M Tris to yield 18,000-22,000 CPM/mL, and 1 mL was added to each tube. Fifty μL of plasma with undetectable PRA was added to all standard tubes to act as a suitable matrix. Tubes were vortexed and incubated at 4°C for 48–72 h to allow the competitive binding of the ^125^I-Ang-I to the anti-angiotensin antibody.

At the end of incubation, 500 μL of cold dextran-coated charcoal in 0.1 M Tris was added to each sample, mixed, and centrifuged. The supernatant was decanted into round-bottom polystyrene tubes (Simport). The supernatants were counted on a Wallac 1260 MULTIGAMMA II gamma counter (Perkin Elmer). Data reduction was performed using StatLIA Enterprise 3.2 (Brendan Technologies). PRA was calculated as ng/L/s using blank subtraction (Equation 1).

(1) $$\mathrm{PRA}\;=\;\left({\left[\mathrm{Ang}‒l\right]}_{37{}^{\circ}C}‒{\left[\mathrm{Ang}‒l\right]}_{4{}^{\circ}C}\right)/\mathrm{\Delta t}$$

### LC-MS/MS procedure

#### Preparation of reference standards

Reference standards were prepared in the same manner as the RIA analysis. Concentrations of 100, 30, 9.0, 2.7, 1.35, 0.675, and 0.3375 ng/mL were prepared in a 1% BSA in 0.1 M Tris buffer.

#### Generation of angiotensin I

Plasma samples were received for LC/ESI-MS/MS analysis in an ice bath, immediately after being aliquoted for RIA analysis. Two hundred and fifty μL of reference standards and plasma samples were added in duplicate to two square polypropylene 2-mL 96 well plates (Corning Inc.), for the 37°C and blank determinations, respectively. The 37°C generation plate contained 50 μL of the generation buffer (1 M Tris, 0.2 M EDTA, and 1 mM PMSF at pH 5.5) resulting in a plasma pH of 6.0. The 37°C generation plate was mixed briefly, and placed in a 37°C water bath for exactly 3 h. The blank plate was extracted by SPE immediately after samples were aliquoted. The blank-subtraction plate was not subjected to a 3-h incubation on ice.

#### Extraction of angiotensin I

Three hundred μL of Ang-I-SIS internal standard, at 10 ng/mL in 10% FA, was aliquoted to each sample, and mixed briefly. A Strata-X 33 μ polymeric reversed phase 60 mg 96 well plate (Phenomenex) was conditioned with 1 mL of methanol followed by 1 mL of 5% FA with vacuum applied at 200 mbar for 1 min for each condition step. The entire sample (550 μL for blank samples and 600 μL for 37°C samples) was loaded onto the plate and flowed through using vacuum applied at 200 mbar for 1 min. The plate was washed with 5% FA followed by 20% methanol with vacuum applied at 200 mbar for 1 min for each wash step and dried under 200 mbar of vacuum for 10 min. Ang-I was eluted from the plate with 250 μL of methanol and collected in a 2-mL polypropylene round-bottom 96 well plate (NUNC/Thermo Fisher) with 200 mbar of vacuum applied for 2 min.

#### LC/ESI-MS/MS analysis

LC/ESI-MS/MS analysis was performed on a UFLC 20 AC (Shimadzu Corporation) with an AB SCIEX API5000 triple-quadrupole (Applied Biosystems). Twenty microliters of sample extracts were injected onto a 50 mm x 2 mm Jupiter 4 μ Proteo 90A Analytical Column (Phenomenex). Mobile phases A and B were 0.2% FA in water, and 0.2% FA in methanol, respectively. Using a 0.5 mL/min flow rate, Ang-I was eluted at 95% B after 0.5 min of column conditioning at 10% B and a gradient to 95% B in 1 min. The system was returned to starting conditions in 0.1 min and re-equilibrated at 10% B for 2.4 min. The total cycle time was 6 min per sample. Triply-charged ions 433.2→647.5 (quantifier) and 433.2→619.3 (qualifier) were monitored for Ang-I and 436.6→657.5 for the SIS-Ang-I internal standard. Calibration curves were fit with 1/*x*^2^ linear regression. PRA was calculated in ng/L/s using Equation 1.

### iMALDI procedure

#### Washing of protein G dynabeads

Protein G Dynabeads were used at a ratio of 5 μL of bead slurry (30 mg/mL) per 1 μg of pAb. Beads were washed 7 times with 1 mL of 25% ACN/1xPBS/0.03% CHAPS, and 3 times with 1xPBS/0.03% CHAPS solution in a 1.5 mL Maxymum Recovery tube (Axygen Scientific). Beads were pelleted with a Dynamag-2 (Invitrogen), allowing 20 s to pellet the beads before removing the supernatant.

#### Conjugation of protein G dynabeads and pAb

Antibody was added to Protein G Dynabeads based on experimental need (1 plate used roughly 23 μg of pAb) at a ratio of 1 μg of pAb to 5 μL of washed beads. A solution of 1xPBS/0.03% CHAPS was added to give a final volume of 7 μL of liquid per 1 μg of pAb. The sample was quickly spun to collect beads at the bottom of the tube and then lightly vortexed to resuspend them. Beads were placed on a Labquake rotor (Fisher Scientific) for 1 h at RT with end-over-end rotation. Following conjugation, beads were pelleted and washed 5 times with 1 mL of 1×PBS/0.03% CHAPS. Once washed, beads were resuspended in 1×PBS/0.03% CHAPS to a pAb concentration of 0.01 μg/μL. Twenty μL of bead solution was transferred to each well of a 96-well skirted PCR plate, which results in 0.2 μg of pAb per capture well.

#### Generation of angiotensin I

Plasma samples were thawed in a RT water bath for 5 min and then placed on ice. Before use, plasma was vortexed and a 200 μL aliquot was added to a 1.5-mL Maxymum Recovery tube, to give a final concentration of 5 mM EDTA, 25 mM maleic acid, 275 μM neomycin trisulphate, and 1 mM PMSF. The final sample pH was 6. The total volume of all inhibitors added was 12.23 μL, resulting in a 6% dilution. Samples were vortexed and split into two tubes containing 100 μL each, representing the blank and generated sample. The blank sample was placed on ice, and the generated sample was placed in a 37°C thermoshake rotor (Eppendorf), shaking at 1000 rpm for exactly 1 h.

#### Antigen capture

Beads were pelleted in the 96 well PCR plate (Axygen) by placing it on a Dynal MPC-96S plate magnet and the supernatant was removed. Tubes were continually vortexed during Ang-I generation, and were then placed on ice. Triplicate analyses of patient blank and generated samples were added to the PCR plate (6 captures per patient). This was repeated for each patient sample, with a total of 13 patients analyzed per plate (78 wells). Triplicate six-point calibration curves at concentrations of 14.8, 7.4, 3.7, 1.9, 0.9, and 0 ng/mL natural Ang-I were prepared in 35 μL of 1×PBS/0.03% CHAPS. Both reference standards and patient samples were spiked with SIS Ang-I internal standard to a concentration of 4.6 ng/mL. After addition of the standards, samples were mixed by pipetting with an 8-channel pipette (Fisher Scientific). The PCR plate was sealed with an Axymat (Axygen), taped to a Labquake rotor, and incubated at 4°C for 1 h with end-over-end rotation.

#### Bead washing and MALDI spotting

Following 1 h of antibody capture, the plate was removed and placed on the PCR plate magnet. Using a multichannel pipette, samples were washed column by column, starting with column 1. The capture solution (plasma and standards) was removed from the pelleted beads. The PCR plate was removed from the magnet and 100 μL of 15% ACN/25 mM ammonium bicarbonate was used to resuspend the beads. The PCR plate was placed back on the magnet and the beads were allowed to pellet for 20 s. This was repeated an additional 2 times for a total of 3 washes. After the final wash, all buffer was removed and the plate was removed from the magnet. Beads were resuspended in 3 μL of 15%ACN/25 mM ammonium bicarbonate, and the entire bead solution was spotted on a MALDI target (part number: 4352802, AB SCIEX). The procedure was performed for each column separately to ensure that the time from first wash to spotting on the MALDI target was consistent across all samples. After all wells had been spotted, the plate was left at RT until all spots were dry and uniform in appearance. Two μL of MALDI matrix solution (containing 3 mg/mL CHCA, 1.8 mg/mL ammonium citrate, 70% ACN, and 0.1% TFA) was overspotted onto each bead spot to elute the peptide and prepare the spot for MALDI analysis.

#### MALDI analysis

Samples were analyzed on an AB SCIEX 4800 MALDI TOF-TOF using Reflector Positive mode, from m/z 800 to 4000. Samples were analyzed in batch mode, after checking before acquisition to ensure that the laser setting produced sufficient intensities for both blank and generated sample. A total of 1250 laser shots were acquired for each spot (10 sub-spectra with 125 shots per sub-spectrum) using a fixed laser intensity. Representative spectra from a 4°C and 37°C capture are shown in Figure 4. Figure 4 also shows a peak at m/z 1295.68 – 1 Da lower than the Natural Ang – which was associated with the antibody. The blank subtraction method for PRA determination removes any potential interference from the ^13^C peak of this component (see Additional file 1).

#### Data analysis and PRA determination

Data was analyzed using AB SCIEX’s Data Explorer software. Peak heights were recorded after performing a baseline correction. Two peak heights were recorded -- those of Natural Ang-I (m/z 1296.68) and SIS Ang-I (m/z 1306.68), and the relative response (RR) was reported as the Nat:SIS ratio. These RR values were compared to the calibration curve. PRA was calculated in ng/L/s by the blank subtraction method (Equation 1). Regressions were performed by the method of Passing and Bablok using cp-R, a graphical user interface to the R statistical programming language [41].

## Abbreviations

RAAS: Renin-angiotensin-aldosterone system; PA: Primary aldosteronism; Ang-I: Angiotensin-I; ACE: Angiotensin-converting-enzyme; Ang-II: Angiotensin-II; PRA: Plasma renin activity; PRC: Plasma renin concentration; iMALDI: Immuno-MALDI; SPE: Solid-phase extraction; SPH: St. Paul’s Hospital; RT: Room temperature; UVic: University of Victoria; FA: Formic acid; SIS: Stable-isotope labelled internal standard; pAb: Polyclonal antibody; CHCA: α-Cyano-4-hydroxycinnamic acid; RR: Relative response; R2: Coefficient of determination.

## Competing interests

The authors declare that they have no competing interests.

## Authors’ contributions

AGC and RP performed the iMALDI experiments; JCvdG performed the LC/MS/MS experiments; DTH and CHB directed the research and interpreted the data; AGC prepared the manuscript. All authors read and approved the final manuscript.

## Authors’ information

Christoph H. Borchers holds the LEEF chair in proteomics in the Department of Biochemistry and Microbiology at the University of Victoria, and he is also director of the University of Victoria - Genome BC Proteomics Centre. Dr. Borchers has a longstanding interest in quantitative proteomics, particularly in moving proteomics into the clinic, and is the developer of the iMALDI technique. Daniel T. Holmes is a Clinical Associate Professor at the University of British Columbia and St. Paul’s Hospital in Vancouver, BC, where his interests are in diagnostic lipidology/endocrinology. Dr. Holmes developed the LC/MS/MS technique for measuring PRA, which was used in this comparative study paper.

## Supplementary Material

Additional file 1 Supplementary information. Click here for file
