# Supplementary material for: Development and evaluation of an immuno-MALDI (iMALDI) assay for angiotensin I and the diagnosis of secondary hypertension
Source: Clin Proteomics. 2013 Dec 20;10(1):20. doi: 10.1186/1559-0275-10-20 (PMC4081661; doi:10.1186/1559-0275-10-20)

**SUPPLEMENTAL INFORMATION**

An interference peak was noticed during the optimization of the iMALDI method. The peak at m/z 1295.68 is 1Da lower than our target Ang-I natural peak (1296.68). The source of this peak was determined to be the antibody, as it is detected in captures containing antibody-bead conjugate and only 1xPBS/0.03% CHAPS buffer. MS/MS analysis identified that the peak was Ang-I with an amidated C-terminus, likely originating from the antibody affinity purification process. The concern was that the ^13^C peak from the 1295.68 m/z contaminant would interfere with our target of interest and affect the calculated PRA for the patient sample.

Supplemental Figure 1A shows the MS/MS spectrum of the 1295.68 m/z peak, in comparison to the MS/MS spectrum of an unmodified Ang-I peptide (Figure 1B). Identification of the b and y ions indicated a -1 Da shift for all the y-ions observed from y_2_ to y_9_, with no change in m/z for the b-ions up to b_9_. This indicates that the modification is on the C-terminus.

We next needed to ensure that the presence of the m/z 1295.68 ion did not affect the PRA assay. Supplemental Figure 2A shows two zoomed regions of a 0^o^C capture (left) and a capture from the same sample generated at 37^o^C for one hour (right). As expected, the m/z 1296.68 peak increases in intensity relative to the Ang-I SIS peak (m/z 1306.68) due to the activity of renin in the patient plasma. Importantly, the m/z 1295.68 peak does not change in intensity during the generation step, and remains at half the intensity of the Ang-I SIS peak. This was further confirmed by comparing the ratio of m/z 1295.68:m/z 1306.68 across 6 replicate samples (3 captures of the 0^o^C sample and 3 captures of the 37^o^C generated sample x 6 samples = 36 data points). The m/z 1295.68:m/z 1296.68 ratios for these 36 data points are shown in Supplemental Figure 2B. The %CV of all of these points is 8.7%. Finally, examination of the m/z 1295.68 peak present in the buffer curve created with each set of measurements indicates that there is no major change in the m/z 1295.68:m/z 1306.68 relative response with increasing Ang-I natural concentration (Supplemental Figure 2C).

In the background subtraction method, the Ang-I concentration determined at 0^o^C is subtracted from the measurement of the Ang-I concentration determined for the sample generated at 37^o^C for 1 hour. Thus only the change in Ang-I concentration between the two samples is important. Because the m/z 1295.68 peak is introduced by the antibody, which is used at a constant amount in all captures, the m/z 1295.68 peak has a constant intensity across samples. Thus, any effect it has on the Ang-I concentration is subtracted from the results by use of the blank subtraction method.

It is important to note that although a peak like this does not present a problem in the PRA assay because it is a "difference" method, it might need to be corrected for in other types of assays. If so, the contribution of an interference peak can be subtracted from the intensity of the target analyte peak. This is a major advantage of mass spectrometric detection over less specific methods.

**Supplemental Figure 1.** A) MS/MS spectrum of 1295.68 m/z peak obtained from an iMALDI reaction containing antibody in 1xPBS/0.03% CHAPS with no peptide present. Fragmentation is nearly identical to the normal pattern observed for Ang-I, with the exception of a -1 Da mass shift for all observed y-ions. The observed b-ions did not have a -1 Da shift. Higher-abundance y- and b-ions are labelled. B) MS/MS spectrum of unmodified Ang-I from a peptide standard solution. All y- and b-ions were detected at their expected m/z values.


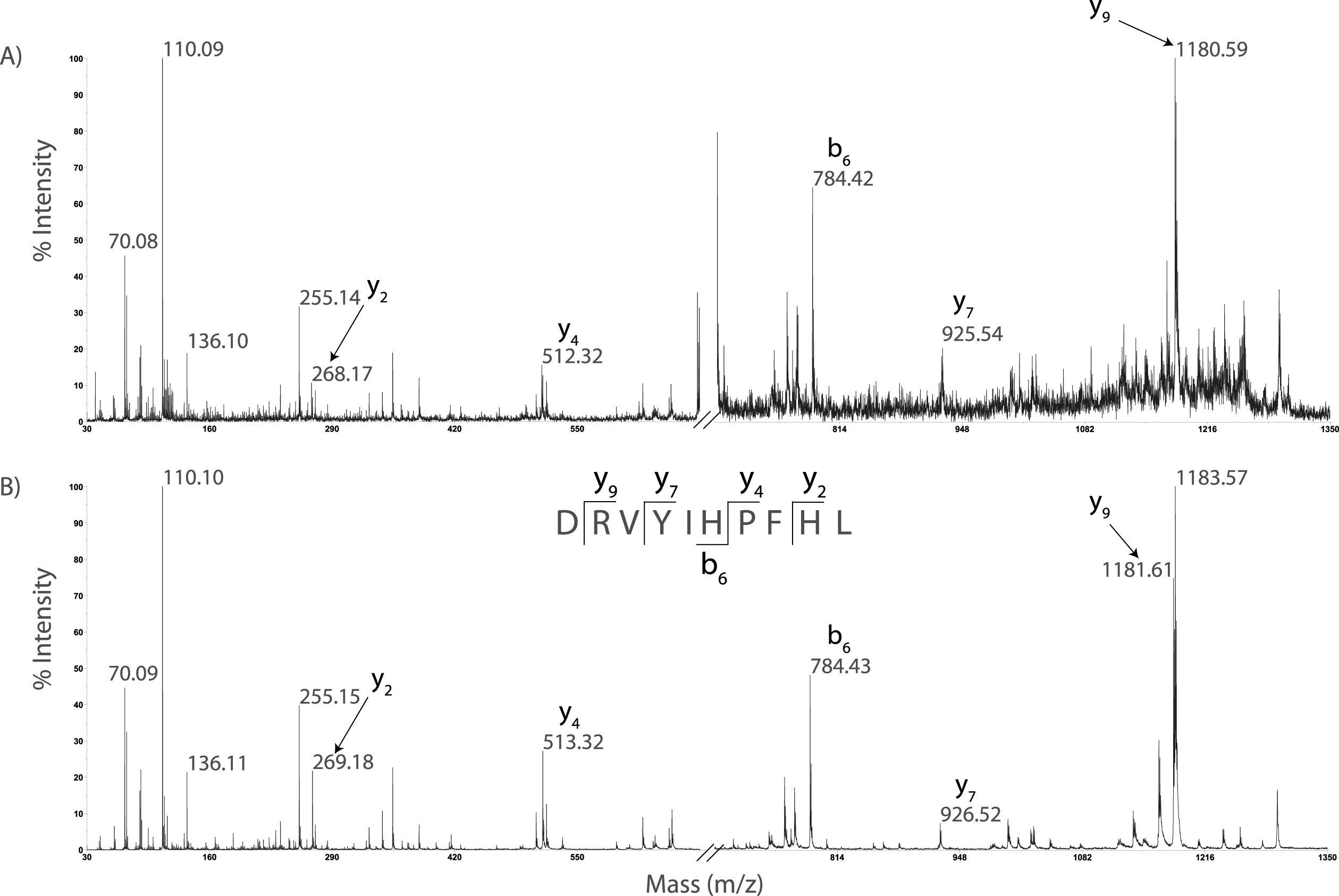


**Supplemental Figure 2.** A) iMALDI captures of patient plasma held at 0^o^C for 1 hour (left) and the same sample generated at 37^o^C for 1 hour (right). The intensity of the natural Ang-I peak (m/z 1296.68) increases relative to the Ang-I SIS peak (m/z 1306.68) due to the activity of renin during the 1 hour generation. The C-terminally amidated Ang-I contaminant peak (m/z 1295.68) does not change in intensity relative to the Ang-I SIS peak. B) 6 plasma samples were analyzed in triplicate for each of the 0^o^C and 37^o^C conditions, and m/z 1295.68: m/z 1306.68 intensities were calculated. The relative response for m/z 1295.68:m/z 1306.68 was constant across all samples, with a CV of only 8.7%. C) Examining the buffer calibration curve for the 1295.68 contaminant peak shows that there is little effect of increasing the Ang-I natural concentration on the relative ratio of the contaminant peak to Ang-I SIS (m/z 1295.68:m/z 1306.68).


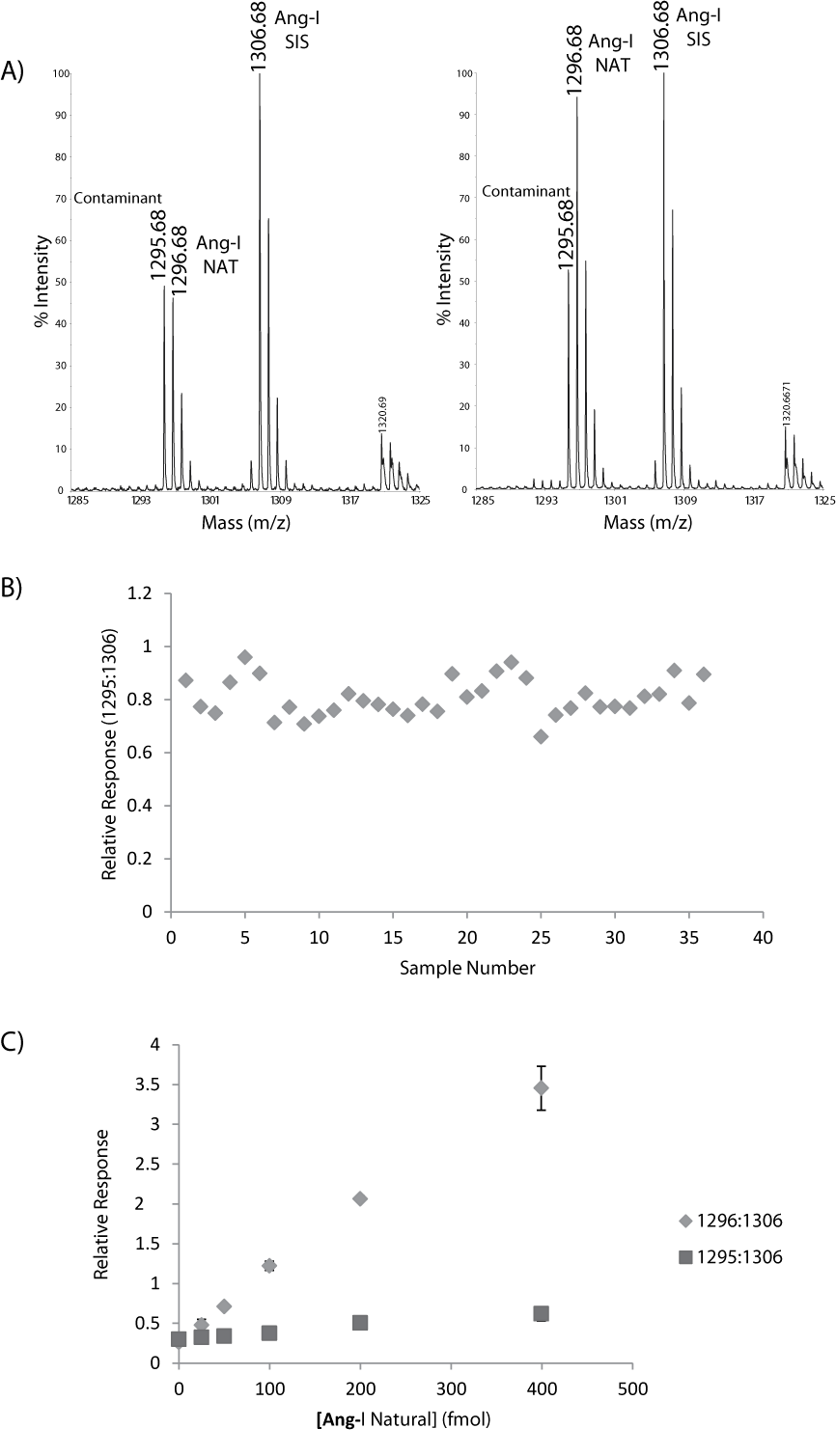

Supplement: Additional file 1 — Supplementary information. [file 1559-0275-10-20-S1.docx]
